# Opiate-use patients attending residential treatment: characteristics, outcomes, and implications for practice

**DOI:** 10.1186/1940-0640-10-S1-A36

**Published:** 2015-02-20

**Authors:** Samuel A MacMaster, Siobhan A Morse, Brian Bride, John Seiters, Cayce M Watson, Sam Choi

**Affiliations:** 1 College of Social Work, University of Tennessee, Nashville, TN, 37210, USA; 2 Foundations Recovery Network in Nashville, Brentwood, TN, 37027, USA; 3 School of Social Work, Georgia State University, Atlanta, GA, 30303, USA; 4 School of Social Work, David Lipscomb University, Nashville, TN, 37204, USA

## Background

As opiate use has increased, there has been a corresponding increase in the number of opiate users presenting for treatment. Questions regarding the challenges of treating opiate users in residential treatment remain largely unanswered. This study seeks to determine what, if any, meaningful differences exist between opiate and non-opiate users, as well as within opiate users who enter voluntary, private, or residential dual-diagnosis treatment, and the impact of any differences relative to treatment motivation, length, and outcomes.

## Materials and methods

Data for this study were drawn from 1972 individuals, utilizing the Addiction Severity Index, the Treatment Service Review, the University of Rhode Island Change Assessment, and a satisfaction measure. Interviews were conducted at program intake, and 1 and 6-month interviews post-discharge.

## Results

The results suggest that although there are similarities there are also some important differences in characteristics, motivation, completion, engagement, retention, levels of satisfaction, and post-treatment service use. Additional analyses were conducted when significant within-group differences by age for opiate users were revealed.

## Conclusions

Results suggest different strategies within treatment programs may provide benefit in targeting the disparate needs of younger opiate users. Outcome results at 6 months for all groups demonstrated significant improvement over pretreatment, suggesting that abstinence-based treatment can be an effective form of treatment for opiate users.

